# Serum p53 antibody detection in patients with impaired lung function

**DOI:** 10.1186/1471-2407-13-62

**Published:** 2013-02-06

**Authors:** Manlio Mattioni, Patrizia Chinzari, Silvia Soddu, Lidia Strigari, Vincenzo Cilenti, Eliuccia Mastropasqua

**Affiliations:** 1 Experimental Research Centre, Regina Elena National Cancer Institute, via delle Messi d’Oro 156, 00158, Rome, Italy; 2 Molecular Oncogenesis Laboratory, Regina Elena National Cancer Institute, via delle Messi d’Oro 156, 00158, Rome, Italy; 3 Medical Physics and Expert Systems Laboratory, Regina Elena National Cancer Institute, via E. Chianesi 53, 00144, Rome, Italy; 4 Respiratory Physiopathology Unit, Regina Elena National Cancer Institute, via E. Chianesi 53, 00144, Rome, Italy

**Keywords:** Impaired lung function, Smoking habits, Serum p53 antibodies, Biomarkers, Lung cancer

## Abstract

**Background:**

*TP53* gene mutations can lead to the expression of a dysfunctional protein that in turn may enable genetically unstable cells to survive and change into malignant cells. Mutant p53 accumulates early in cells and can precociously induce circulating anti-p53 antibodies (p53Abs); in fact, p53 overexpression has been observed in pre-neoplastic lesions, such as bronchial dysplasia, and p53Abs have been found in patients with Chronic Obstructive Pulmonary Disease, before the diagnosis of lung and other tobacco-related tumors.

**Methods:**

A large prospective study was carried out, enrolling non-smokers, ex-smokers and smokers with or without the impairment of lung function, to analyze the incidence of serum p53Abs and the correlation with clinicopathologic features, in particular smoking habits and impairment of lung function, in order to investigate their possible role as early markers of the onset of lung cancer or other cancers. The p53Ab levels were evaluated by a specific ELISA in 675 subjects.

**Results:**

Data showed that significant levels of serum p53Abs were present in 35 subjects (5.2%); no difference was observed in the presence of p53Abs with regard to age and gender, while p53Abs correlated with the number of cigarettes smoked per day and packs-year. Furthermore, serum p53Abs were associated with the worst lung function impairment. The median p53Ab level in positive subjects was 3.5 units/ml (range 1.2 to 65.3 units/ml). Only fifteen positive subjects participated in the follow-up, again resulting positive for serum p53Abs, and no evidence of cancer was found in these patients.

**Conclusion:**

The presence of serum p53Abs was found to be associated with smoking level and lung function impairment, both risk factors of cancer development. However, in our study we have not observed the occurrence of lung cancer or other cancers in the follow-up of positive subjects, therefore we cannot directly correlate the presence of serum p53Abs with cancer risk.

## Background

Lung cancer is the leading cause of cancer death in the world; in the United States, the death rate is 26% in women and 31% in men [1]. Despite improved therapy, the survival outcome is often limited by late diagnosis, when lung cancer is inoperable, and overall 5-year survival is only 15%. It is, therefore, necessary to search for new diagnostic tools to identify lung cancer in the early stages. Mutations in the *TP53* tumour suppressor gene are the most common genetic alterations in human cancers [2] and most can lead to the expression of mutant p53 proteins with a half-life longer than for the wild type, which then accumulate in cancer cells. Accumulation has also been found in pre-neoplastic lesions and normal tissues surrounding the tumours, suggesting that it occurs early on in cancer progression [3,4]. The accumulation of p53 can in turn induce circulating anti-p53 antibodies (p53Abs), and in fact there is a close correlation between serum p53Abs and p53 overexpression in the corresponding tissues [5], so that p53Abs can be considered as early markers for the presence of p53 mutations. Indeed, serum p53Abs were found in patients with Barrett’s metaplasia of the oesophagus evolving into dysplasia and cancer as a consequence of chronic reflux; p53 accumulation especially occurs during transition from low to high grade dysplasia and the appearance of p53Abs may predate the diagnosis of oesophageal carcinoma [6]. These antibodies have also been showed in serum of patients with ulcerative colitis, at high risk of developing colon cancer, and their presence was regarded as an early marker of malignant progression [7]. Further, serum p53Abs were detected in workers occupationally exposed to asbestos, at high risk of cancer, before any clinical evidence of malignancy [8]. Altogether, these data suggest that serum p53Abs may have predictive value for the subsequent development of cancer. In lung cancer, in particular, p53 mutations arise early on, since p53 accumulation was detected in pre-neoplastic lesions such as bronchial dysplasia [9] and serum p53Abs were found in isolated cases of both heavy smokers and patients with Chronic Obstructive Pulmonary Disease (COPD), at high risk of lung and other tobacco-related cancers, several months before the diagnosis of cancer [10,11].

Further, a correlation between tobacco smoking and lung cancer has been demonstrated [12,13] and several studies have shown increased risk of lung cancer in patients with COPD [14,15], in particular for the squamous histological subtype [16]. Cigarette smoking is the main aetiological factor of both COPD and lung cancer, since cigarette smoke contains elevated concentrations of oxidants and carcinogens that can induce persistent lung inflammation and mutations [17]. Chronic inflammation has been demonstrated to play a central role in cancer pathogenesis [18] and recent studies have linked Nuclear Factor (NF)-kB, major mediator of inflammation, to carcinogenesis [19]. p53 can suppress inflammatory response by inhibiting NF-kB activity [19] and since it is often mutated by cigarette smoke, oxidant activation of NF-kB may result in a chronic imbalance in COPD and lung cancer. In addition, p53 can reduce COX-2 expression [20], another inflammatory mediator involved in lung cancer development and progression [21], and loss of p53 activity may contribute to the persistent elevation of COX-2 in epithelial stroma and lung cancer cells. Furthermore, COPD frequently shows squamous metaplasia with dysplastic areas at different bronchial levels; metaplasia has been correlated with the response to chronic inflammation and is associated with p53 mutations [22]. Finally, an increased risk of lung cancer has also been reported in patients with restrictive lung disease [23], only slightly associated with tobacco smoking, in which inflammation of the lung may independently contribute to the pathogenesis of lung cancer.

Therefore, the aim of our work was to investigate, in a large prospective study, the incidence of p53Abs, biomarkers of p53 mutations, in heavy smokers and patients with impaired lung function, at high risk of lung cancer and other cancers, in order to evaluate their relationship with tobacco smoke exposure and chronic airflow limitation, in view of a possible role in the early diagnosis of cancer.

## Methods

### Patients

A total of 675 people, including 399 subjects with normal lung function tests (214 men, 185 women; median age 56 years, range 26 to 93 years; 42 non-smokers, 88 ex-smokers, 269 current smokers) and 276 patients with obstructive or restrictive type lung function tests (169 men, 107 women; median age 62 years, range 24 to 83 years; 44 non-smokers, 80 ex-smokers, 152 current smokers; 152 with mild, 73 with moderate and 51 with severe impairment of lung function tests), were evaluated (Table 1). All people were recruited at the Respiratory Physiopathology Unit of the Regina Elena National Cancer Institute, Rome, Italy, between June 2004 and March 2009. They were enrolled either by a voluntary pulmonary visit at the Respiratory Physiopathology Unit or coming from the Tumour Prevention Centre of the same Institute, because of increased risk of lung cancer. Regular smokers, ex-smokers and non-smokers were included, without age limit, or history of previous malignant diseases. The number of non-smokers was small compared to smokers, because they were only recruited at the Respiratory Physiopathology Unit along with smokers and ex-smokers, while only the latter groups, associated with lung cancer risk, were taken from the Tumour Prevention Centre. Tobacco smoke exposure was further evaluated as number of cigarettes smoked per day, years of tobacco smoking and, in ex-smokers, number of years since quitting. Ex-smokers were defined as people that had quit smoking at least 6 months prior to the lung function tests. All the people enrolled received a routine physical examination and underwent lung function tests and low dose CT scans of the chest at the start of the study. Serum samples were obtained from all subjects at the time of the lung function tests, thereafter, fifteen people positive for serum p53Abs, who complied with the follow-up until June 2011, were tested for serum p53Ab levels with a median follow-up of 24 months (range 6 to 60 months) and further evaluated by CT scans of the chest, specialists or other instrumental examinations suitable for monitoring the development of lung cancer or other cancer types, such as breast, prostate or colon cancers. The study was approved by the ethical committee on human experimentation of the promoting institution, Regina Elena National Cancer Institute, Rome, Italy. All participants in the study gave their written informed consent.

**Table 1:** Patient characteristics

	NORMAL LFT*	**IMPAIRED LFT**
	399	276
**Age**		
Median (range), years	56 (26–93)	62 (24–83)
**Gender**		
Male	214 (54%)	169 (61%)
Female	185 (46%)	107 (39%)
**Smoking habit**		
Non-smokers	42 (11%)	44 (16%)
Current smokers	269 (67%)	152 (55%)
Ex-smokers	88 (22%)	80 (29%)
*No*. *of cigarettes smoked per day*		
≤ 20	241 (60%)	129 (47%)
> 20	114 (29%)	102 (37%)
Missing	2	1
*Packs*-*year*		
≤ 40	239 (60%)	117 (42%)
> 40	116 (29%)	113 (41%)
Missing	2	2
*Years since quitting*		
≤ 10	35 (9%)	37 (13%)
>10	29 (7%)	25 (9%)
Missing	24	18
**LFT impairment** (obstructive or restrictive)		
Mild		152 (55%)
Moderate		73 (26%)
Severe		51 (18%)

* LFT, Lung Function Tests.

### Serum p53Ab assay

All serum samples were aliquoted, coded, and stored at −80°C until assays were performed. p53Abs were detected by a commercially available, highly specific ELISA kit (Anti-p53 ELISA II kit, PharmaCell, Paris, France), using micro-titre plates coated either with human recombinant p53 protein to detect specific p53Abs, or with control proteins to reveal non-specific interactions. The assay was performed according to the manufacturer’s instructions. All samples were tested blindly, twice in the same assay. Absorbance was measured at 450 nm and 620 nm, using a programmable ELISA reader. p53Ab levels ≥ 1.2 unit/ml were considered as positive, according to the manufacturer’s suggestion; this cut-off was in agreement with other studies [24].

Usually, p53Abs recognize epitopes in the amino and carboxyl termini of p53 protein, outside the DNA-binding domain where most mutations occur, thus identifying both wild type and mutant p53 proteins; however, p53Abs to certain types of mutant p53 proteins might not bind to the wild type recombinant p53 protein used as antigen in our assay, with the possibility of false negative results. Furthermore, ELISA shows a high specificity, but a low sensitivity for serum antibody detection and novel and more sensitive methods have been developed, such as the particle agglutination assay [25]. However, ELISA still retains its value for diagnostic accuracy and easy performance in routine diagnostic procedures [26].

### Lung function tests

All people were subjected to lung function tests by two spirometers (Altair 1000 and Quark PFT). Lung function tests were carried out according to the American Thoracic Society evaluation methods and the values were reported as percentages of the following parameters: total lung capacity (TLC), forced vital capacity (FVC), forced expiratory volume at first second (FEV1) and FEV1/FVC ratio (Tiffenau index).

Within pulmonary diseases, two types of ventilation defects are identified by lung function tests: obstructive or restrictive type. An obstructive defect of pulmonary ventilation is defined by a Tiffenau index <70%, while stage of obstruction is specified by the FEV1 value as follows: mild, FEV1 ≥70%; moderate, FEV1 <70% but ≥60%; moderately severe, FEV1 <60% but ≥50%; severe, FEV1 <50% but ≥34%; very severe, FEV1 <34%. After evaluation of these parameters, patients were divided into three groups: with mild, moderate or severe (including moderately severe, severe, and very severe) obstructive defects.

A restrictive defect of pulmonary ventilation is characterized by TLC reduction; according to this index, the restrictive defect has been distinguished as: mild, TLC >70%; moderate, TLC from 70% to 60%; moderately severe, TLC <60% but >50%; severe, TLC from 50% to 34%; very severe, TLC <34%. After evaluation of this parameter, patients were divided into three groups: with mild, moderate or severe (including moderately severe, severe, and very severe) restrictive defects.

### Statistical analysis

All statistical analyses were performed by using R [27]. Independent variables were first evaluated for unconditional associations with the dependent variable using a chi-square test for categorical data and t test for continuous data. If a continuous variable did not satisfy the normality assumption, the Wilcoxon rank-sum test was used. Correlations between independent continuous variables were assessed based on Pearson’s correlation coefficient for the normally distributed variables. Correlations between variables that did not satisfy the normality condition were assessed based on Spearman’s rho coefficient. Associations between independent categorical and continuous variables were assessed by the t test, the Wilcoxon rank-sum test, or the exact Wilcoxon rank-sum test, as appropriate. Multivariate analysis was performed on the variables, resulting statistically significantly associated in the univariate analysis. In the univariate/multivariate analysis, P values of <0.05 were considered statistically significant.

## Results

Sera from 675 subjects, including non-smokers, ex-smokers and current smokers, with normal or impaired lung function tests, were evaluated for the presence of p53Abs. Our results showed that 5.2% of them (35 out of 675) had significant levels of serum p53Abs, with a median value of 3.5 units/ml (range 1.2 to 65.3 units/ml). No difference was found by age or gender. An association between serum p53Abs and smoking habit has been observed, although not statistically significant: two out of 86 non-smokers (2.3%), 23 out of 421 current smokers (5.5%) and ten out of 168 ex-smokers (6.0%) were positive for p53Abs; furthermore, in subjects with normal lung function tests, none of the 42 non-smokers (0%), fifteen out of 269 current smokers (5.6%) and six out of 88 ex-smokers (6.8%) had p53Abs, while in patients with impaired tests, two of the 44 non-smokers (4.6%), eight out of 152 current smokers (5.3%) and four out of 80 ex-smokers (5.0%) resulted p53Ab positive, suggesting that the association of serum p53Abs with smoking habit was more evident in cases of normal lung function. Airway inflammation has a part also in lung function decline independent of smoking, as observed in asthma and pulmonary fibrosis. Through oxidative DNA damage, this inflammation can promote p53 mutation and overexpression in the surrounding lung cells, with subsequent induction of serum p53Abs; this might be the reason why we found two p53Ab positive patients amongst the non-smokers with impaired lung function. A statistically significant correlation between the rate of p53Ab positive subjects and the number of cigarettes smoked per day was found (p = 0.019), while a trend of increase with packs-year was observed (p = 0.092, Table 2).

**Table 2:** Correlation between serum p53Abs and clinicopathologic parameters

		**p**-**value**^**a**^
**Age** (years)	(< 58 / ≥ 58)	
Negative	308 / 328 (48% / 52%)	0.502
Positive	19 / 15 (56% / 44%)	
Missing	5	
**Gender**	Male / Female	
Negative	359 / 281 (56% / 44%)	0.202
Positive	24 / 11 (69% / 31%)	
**No**. **of cigarettes per day**	(> 20 / ≤ 20)	
Negative	197 / 356 (36% / 64%)	0.019*
Positive	19 / 14 (58% / 42%)	
**Packs**-**year**	(> 40 / ≤ 40)	
Negative	211 / 341 (38% / 62%)	0.092^#^
Positive	18 / 15 (55% / 45%)	
**LFT impairment**	Moderate-Severe / Normal-Mild	
Negative	113 / 527 (18% / 82%)	0.068^#^
Positive	11 / 24 (31% / 69%)	

^a^Calculated by chi-square test;* p < 0.05, ^#^ p < 0.10.

We then considered the correlation between serum p53Abs and impairment of lung function of either the obstructive or restrictive type, classified as mild, moderate, and severe. Twenty one of the 399 subjects with normal lung function tests (5.3%) and fourteen of the 276 patients with altered tests (5.1%) were positive for p53Abs. However, in the latter group, three out of 152 patients with mild (2.0%), eight out of 73 with moderate (11.0%) and three out of 51 with severe (5.9%) impairment of lung function had p53Abs. Then, we examined the correlation between serum p53Abs and, on one hand, subjects with normal lung function or mildly altered tests and, on the other hand, patients with moderate to severe impairment of lung function. A higher rate of serum p53Abs was found as a trend (p = 0.068) in patients with moderate to severe impairment of lung function tests, in comparison to subjects with normal or mildly altered tests (Table 2), this correlation was confirmed by a multivariate analysis (p = 0.045). Furthermore, the multivariate analysis showed the lung function test impairment to be a statistically significant predictor of serum p53Ab detection, but not other parameters, such as age, gender or smoking habit.

We further investigated whether there were differences in the presence of serum p53Abs between people with normal lung function tests and patients with altered lung function tests with regard to age, gender, number of cigarettes per day and packs-year (Table 3). No difference was found by age or gender; on the other hand, there was a significant correlation of serum p53Abs with cigarettes smoked per day (p = 0.001) and an increased trend with packs-year (p = 0.081) for the normal lung function group, while no correlation was observed in patients with altered lung function tests.

**Table 3:** **Correlation between serum p53Abs and clinicopathologic parameters**, **distinguishing between subjects with normal or altered LFT**

	**Normal LFT**	**p**-**value**^**a**^	**Altered LFT**	**p**-**value**^**a**^
**Age** (years)	(< 58 / ≥ 58)		(< 58 / ≥ 58)	
Negative	221/154 (59%/ 41%)	0.233	87/174 (33%/67%)	0.938
Positive	15/5 (75%/25%)		4/10 (29%/71%)	
Missing	4		1	
**Gender**	Male / Female		Male / Female	
Negative	202/176 (53%/47%)	0.915	157/105 (60%/40%)	0.136
Positive	12/9 (57%/43%)		12/2 (86%/14%)	
**No**. **of cigarettes per day**	(> 20 / ≤ 20)		(> 20 / ≤ 20)	
Negative	100/234 (30%/70%)	0.001*	97/122 (44%/56%)	0.904
Positive	14/7 (67%/33%)		5/7 (42%/58%)	
**Packs**-**year**	(> 40 / ≤ 40)		(> 40 / ≤ 40)	
Negative	105/229 (31%/69%)	0.081^#^	106/112 (49%/51%)	0.720
Positive	11/10 (52%/48%)		7/5 (58%/42%)	

^a^Calculated by chi-square test; * p < 0.05, ^#^ p < 0.10.

Further, median serum p53Ab levels were calculated to evaluate whether there was any correlation with clinicopathologic features such as age, gender, smoking status and lung function impairment, but no correlation was found as reported in Table 4. With regard to quitting smoking, no significant difference in p53Ab levels was observed over time, presumably due to the small number of positive subjects; in fact, four positive subjects were found out of 72 ex-smokers, who had quit smoking for ≤10 years, and three positive subjects out of 54 ex-smokers, who had quit for >10 years, with a median serum p53Ab level of 4.1 and 3.9 units/ml, respectively.

**Table 4:** Correlations between median serum p53Ab levels and clinicopathologic parameters

	**Median serum p53Ab levels** (**units**/**ml**)	**p**-**value**^**a**^
**Age** (years)	(< 58 / ≥ 58)	
	3.5 / 3.6	0.510
**Gender**	Male / Female	
	3.55 / 2.5	0.582
**No**. **of cigarettes per day**	(> 20 / ≤ 20)	
	3.5 / 2.5	0.232
**Packs**-**year**	(> 40 / ≤ 40)	
	3.55 /2.5	0.143
**LFT impairment**	Moderate-Severe / Normal-Mild	
	5.0 / 8.0	0.371
**LFT impairment**	Mild to Severe / Normal	
	4.6 / 7.8	0.285

^a^Calculated by chi-square test.

Finally, due to follow-up loss, only fifteen subjects positive for serum p53Abs were further assessed for the presence of these Abs, with a median follow-up of 24 months (range 6 to 60 months), again resulting positive for p53Abs, and no one has shown any sign of incipient cancer by CT of the chest or other specific examinations, carried out to verify the development of lung cancer or other cancers, such as breast, prostate or colon cancers.

## Discussion

In the present investigation, we detected significant levels of serum p53Abs in 35 (5.2%) out of 675 subjects, including non-smokers, ex-smokers and current smokers, with normal or impaired lung function tests. With regard to smoking status, a trend, although not statistically significant, was found in the frequency of p53Abs, increasing from non-smokers (2.3%) to current smokers (5.5%) and ex-smokers (6.0%); the differences were more evident in subjects with normal lung function tests, while nil in patients with impaired tests. Further, we showed a significant correlation between the presence of serum p53Abs and the number of cigarettes smoked per day, while an increased trend between p53Abs and packs-year was observed. Furthermore, a higher rate of p53Ab positive sera was found as a trend in patients with moderate to severe impairment of lung function, compared to subjects with normal or mildly altered lung function. We also considered the difference in serum p53Abs between subjects with normal lung function and patients with altered lung function with regard to the number of cigarettes smoked per day and packs-year and found a significant correlation of p53Abs with cigarettes smoked per day and an increased trend with packs-year in the normal lung function group, while no correlation was observed in patients with altered lung function tests. Finally, none of the follow-up p53Ab positive subjects showed development of lung cancer or other cancers, such as breast, prostate and colon cancers.

Cigarette smoking is closely correlated with p53 mutations. Husgafvel-Pursiainen and co-authors [28] observed that the frequency of p53 mutations increased from non-smokers to ex-smokers, reaching the highest rate in current smokers. Li and co-authors [29] reported a similar trend with frequency of serum p53Abs that increased from non-smokers to ex-smokers and current smokers, with heavy smokers having the highest prevalence. Our study also shows that current smokers (5.5%) and ex-smokers (6.0%) have higher frequencies of serum p53Abs than non-smokers (2.3%). Lubin and co-authors [10] and Trivers and co-authors [11] found that serum p53Abs can be detected in ex-smokers and current smokers even 15 months before the diagnosis of lung, breast, and prostate cancers, suggesting that serum p53Abs, closely associated with p53 mutations, may be useful in the early diagnosis of tobacco-related cancers.

Impaired lung function has also been associated with an increased risk of lung cancer. In a meta-analysis, Wasswa-Kintu and co-authors [14] observed that, independent of cigarette smoking, reduced FEV1 increased lung cancer risk in the general population; in addition, patients with the worst lung function showed the highest risk, while subjects with normal lung function had the lowest risk. Furthermore, even small differences in FEV1 significantly increased the risk of lung cancer; finally, the risk was amplified in women. In a very large prospective study, Purdue and co-authors [23] found an increased risk of lung cancer in patients with either obstructive or restrictive impairment of lung function. In our investigation, a higher rate of serum p53Abs was found in patients with the worst lung function alterations of either obstructive or restrictive type, thus confirming that p53Abs may be associated with patients at increased risk of lung cancer. Impaired lung function may derive through conditions that increase the risk of lung cancer, such as inflammation of the airways, which plays a role in smokers and patients with asthma or COPD [30]; on the other hand, inflammatory processes responsible for lung restriction may also contribute to lung cancer pathogenesis [31]. Since p53 can function as an inhibitor of inflammation [19], mutant p53 proteins may be involved in deregulated inflammation contributing to the pathogenesis of lung cancer and other cancers and then serum p53Abs may be early markers of tumor development in people at high risk of cancer, such as patients with impaired lung function. However, since this study shows there was only a small number of p53Ab positive subjects who complied with the follow-up, and neither lung cancer nor other cancers were observed, we cannot correlate the presence of serum p53Abs with cancer risk.

Other well defined markers of lung cancer are the Kirsten rat sarcoma viral oncogene homolog (KRAS) and Epidermal growth factor receptor (EGFR). KRAS is involved in several signalling pathways and mutations in this gene may lead to cancer development. In fact, KRAS mutations are present up to 30% in non-small cell lung cancers (NSCLC). They are found prevalently on codon 12 and appear early in cancer development; furthermore, in some tumors they have been detected in blood before clinical diagnosis [32]. On the other hand, EGFR is highly expressed in various cancers, including lung cancer. EGFR is a member of the family of EGF tyrosine kinase receptors and upon ligand binding activates several intracellular pathways. A soluble fragment of the EGFR extracellular ligand domain can be detected by ELISA in the blood of cancer patients, including NSCLC patients, and may also be elevated at an early stage of carcinogenesis in asbestosis patients [33]. Thus, KRAS and EGFR might have a role as markers of lung function impairment that may reflect cancer risk. Of interest, other proteins can be used to detect lung function impairment, such as Fibrinogen, Neutrophil gelatinase associated lipocalin, Extracellular newly identified RAGE-binding protein and Heparin-binding EGF-like growth factor, showing significantly different serum levels when comparing mild/moderate and severe/very severe COPD patients to smoking and non-smoking controls [34].

## Conclusions

Detection of serum p53Abs in people at high risk of lung cancer and other cancers, such as heavy smokers and patients with impaired lung function, shows a correlation with cigarettes smoked per day, packs-year and the worst impairment of lung function tests. However, in our study, no correlation was observed between serum p53Abs and cancer risk.

## Abbreviations

ELISA: Enzyme-Linked Immunosorbent Assay; CT: Computed Tomography; p53Abs: Anti-p53 antibodies; COPD: Chronic Obstructive Pulmonary Disease; NF-kB: Nuclear Factor-kB; COX-2: Cyclooxygenase-2; TLC: Total Lung Capacity; FVC: Forced Vital Capacity; FEV1: Forced Expiratory Volume at first second; KRAS: Kirsten rat sarcoma viral oncogene homolog; EGFR: Epidermal growth factor receptor; NSCLC: Non-small cell lung cancer; RAGE: Receptor for advanced glycation end products.

## Competing interests

The authors declare that they have no competing interests.

## Authors’ contributions

MM study design, data interpretation, manuscript preparation; PC immunoassay performance, data interpretation; SS data interpretation, manuscript preparation; LS statistical analysis, manuscript preparation; VC study design, data interpretation, manuscript preparation; EM data collection, data interpretation. All authors read and approved the final manuscript.

## Pre-publication history

The pre-publication history for this paper can be accessed here:

http://www.biomedcentral.com/1471-2407/13/62/prepub
